# Disrupting and diversifying the values, voices and governance principles that shape biodiversity science and management

**DOI:** 10.1098/rstb.2022.0196

**Published:** 2023-07-17

**Authors:** Anne K. Salomon, Daniel K. Okamoto, Ḵii'iljuus Barbara J. Wilson, hiininaasim Tommy Happynook, Wickaninnish, wiicuckum Anne Mack, Skil Hiilans Allan Davidson, Gidansda Guujaaw, Wigvilhba Wakas Harvey L. Humchitt, Tom Mexsis Happynook, weiwimtaeek Christina Cox, Hyuuštulth Francis Gillette, n'yasim Samantha Christiansen, Dianna Dragon, Hannah M. Kobluk, Lynn C. Lee, M. Tim Tinker, Jennifer J. Silver, Derek Armitage, Iain McKechnie, Aaron MacNeil, Dylan Hillis, Ella-Kari Muhl, Edward J. Gregr, Christian J. C. Commander, Arianna Augustine

**Affiliations:** ^1^ School of Resource and Environmental Management, Simon Fraser University, 8888 University Drive, Burnaby, British Columbia, Canada V5A 1S6; ^2^ Department of Biological Science, Florida State University, 319 Stadium Drive, Tallahassee, FL 32303, USA; ^3^ St'awaas Xaaydaga, Ruling Eagle Clan, Cumshewa, Haida Gwaii, British Columbia, Canada V0T 1S0; ^4^ Department of Anthropology, University of Victoria, PO Box 1700 STN CSC, Victoria, British Columbia, Canada V8W 2Y2; ^5^ PO Box 1329 Port Alberni, British Columbia, Canada V9Y ZM2; ^6^ 'tuk^w^aaʔatḥ Nation, Macoah, British Columbia, Canada V0R 3A0; ^7^ Haida Nation, Old Masset, Haida Gwaii, British Columbia, Canada V0T 1M0; ^8^ Haida Nation, Skidegate, Haida Gwaii, British Columbia, Canada V0T 1S1; ^9^ Heiltsuk Nation, Bella Bella, British Columbia, Canada V0T 1Z0; ^10^ Huu-ay-aht Nation, Anacla, British Columbia, Canada V0R 1B0; ^11^ Ka:'yu:'k't'h’ Nation, Kyuquot, British Columbia, Canada VOP 1J0; ^12^ Che:k:tles7et'h’ Nation, Kyuquot, British Columbia, Canada VOP 1J0; ^13^ Gwaii Haanas National Park Reserve, National Marine Conservation Area Reserve, and Haida Heritage Site, 60 Second Beach Road, Skidegate, British Columbia, Canada V0T 1S1; ^14^ Nhydra Ecological Consulting, 11 Parklea Drive, Head of St Margarets Bay, Nova Scotia, Canada B3Z 2G6; ^15^ Geography, Environment and Geomatics, University of Guelph, 50 Stone Road East, Guelph, Ontario, Canada N1G 2W1; ^16^ School of Environment, Resources and Sustainability, University of Waterloo, 200 University Ave W, Waterloo, Ontario, Canada N2L 3G1; ^17^ Ocean Frontier Institute, Department of Biology, Dalhousie University, Halifax, Nova Scotia, Canada B3H 4R2; ^18^ Department of Mathematics and Statistics, Dalhousie University, Halifax, Nova Scotia, Canada B3H 4R2; ^19^ Institute for Resources Environment, and Sustainability, University of British Columbia, 2202 Main Mall, Vancouver, British Columbia, Canada V6T 1Z4; ^20^ Scitech Environmental Consulting 2136 Napier St., Vancouver, British Columbia, Canada V5L 2N9; ^21^ Stz'uminus Nation, 1041-B Trunk Rd, Duncan, British Columbia, Canada V9L 2S4

**Keywords:** biodiversity, equity, environmental justice, governance, sea otters, kelp forest

## Abstract

With climate, biodiversity and inequity crises squarely upon us, never has there been a more pressing time to rethink how we conceptualize, understand and manage our relationship with Earth's biodiversity. Here, we describe governance principles of 17 Indigenous Nations from the Northwest Coast of North America used to understand and steward relationships among all components of nature, including humans. We then chart the colonial origins of biodiversity science and use the complex case of sea otter recovery to illuminate how ancestral governance principles can be mobilized to characterize, manage and restore biodiversity in more inclusive, integrative and equitable ways. To enhance environmental sustainability, resilience and social justice amid today's crises, we need to broaden who benefits from and participates in the sciences of biodiversity by expanding the values and methodologies that shape such initiatives. In practice, biodiversity conservation and natural resource management need to shift from centralized, siloed approaches to those that can accommodate plurality in values, objectives, governance systems, legal traditions and ways of knowing. In doing so, developing solutions to our planetary crises becomes a shared responsibility.

This article is part of the theme issue ‘Detecting and attributing the causes of biodiversity change: needs, gaps and solutions’.

According to the theory of **tsawalk** (one), any planetary stage of crisis must, by definition, be a shared responsibility, a shared experience … To this day, responsibility for the planet has not been shared. Umeek Richard Atleo, 2011 [1, pp. 57–58].

## 1. Introduction

The sciences face a reckoning in this moment of time defined by a rapidly changing climate, accelerating exploitation of Earth's biosphere, and increasing recognition of the role science plays in generating and institutionalizing inequity among people [2]. As a society, we are grappling with the systems of power that shape the aims, impacts and beneficiaries of the sciences tackling these global crises [3–5]. Consequently, the links between biodiversity, environmental change, equity and justice have come into sharp focus in local to global policy dialogues and scientific initiatives aiming to support them. At the heart of these introspections are questions surrounding the worldviews and associated values that motivate science, influence environmental decision making, and ultimately, determine who benefits and how.

Values are individually and collectively held evaluative beliefs that inform preferences for different states of being, ways of understanding and courses of action [6]. Values underlie governance principles, laws, policies and management actions. Values also shape the direction, funding and motivations for scientific inquiry and influence if and how basic science principles are adopted, applied and enshrined in institutions and practices. Yet, values are often unstated, unrecognized, assumed or overlooked. For example, the sciences that seek to quantify and understand biodiversity tend to be predicated on Eurocentric values, voices, and knowledge systems where people are considered external disruptors to and beneficiaries of biodiversity, rather than prominent components of it. Moreover, the management of biodiversity on land and in the sea tends to reflect individualistic and instrumentalist values such that populations of non-human species are termed ‘stocks’ or ‘natural resources’ available primarily for exploitation. Consequently, Western definitions and views of biodiversity hold fundamental assumptions that are largely unrecognized and thus unchallenged. While contemporary biodiversity science, laws, governance structures and management actions have an important role to play in environmental and species-at-risk decision-making, they tend to centre values that are aligned with neoliberal and colonial systems of power [7] and are thereby focused on a narrow set of policy objectives serving a narrow set of interests.

The need to broaden the values that inform contemporary biodiversity science and management is pressing, as is the need to widen our consideration of social–ecological relationships and baselines that are generally considered normative or widely agreed-upon. Millennia of reciprocal interactions between people and place throughout the Holocene have informed values that shape Indigenous governance principles, systems and management innovations, developed and honed over the long-term. In many cases these governance principles and systems consider humans as integral to ecosystems [8], and as such, see people as having a valuable role to play in enhancing biodiversity and the sustainability and resilience of societies [9,10].

In this essay, we begin by describing shared governance principles of 17 First Nations along the Pacific Coast of North America, developed since time immemorial to understand, describe and manage relationships among all components of nature, including humans. Principles are explicit expressions of values, in this case those that shape knowledge systems, laws and management practices. Next, we trace the relatively brief 250-year history of biodiversity science, the extractive value systems that shaped its origins, and how it has become institutionalized in some management applications and omitted from others. We then use the example of Canada's Pacific kelp forests and the recovery of a once endangered keystone predator, *kuu (Xaayda Kil),* K̓ʷak̓ʷaƛ *(nuučaan̓uł), q̓asá (Haíɫzaqv), Enhydra lutris (Latin binomial*), henceforth sea otters, to being to advance and understanding of how Indigenous values, governance principles, and laws can be used in practice to characterize, monitor and manage biodiversity change broadly in more inclusive, integrative, and equitable ways.

### (a) Positionality

We are a collective of hereditary leaders, Indigenous knowledge-holders, researchers, practitioners and artists applying Indigenous and Western knowledge and methodologies to inform conservation policies and governance arrangements that better reflect the social-ecological interactions and diverse values held by people along the Northwest Coast of North America. Collectively, we address interrelated questions on species recovery, fisheries management and food security amid climate uncertainty, and the negotiation of reconciliation between Indigenous and colonial governments. The Indigenous knowledge-holders among us represent hereditary leaders of the nuučaan̓uł (anglicized to Nuu-chah-nulth), Haíɫzaqv (anglicized to Heiltsuk) and Xaayda (anglicized to Haida) Nations, original and contemporary sovereign governments in this region responsible for managing the relationships between people, lands and waters prior to the incursion of settler-colonial laws and actively reclaiming that authority today. The researchers among us represent diverse disciplines, from marine ecology and fisheries science to anthropology and political ecology, each reflecting a distinct epistemology. All of us recognize the privilege and responsibility we hold in society broadly and in environmental decision-making specifically. Our diverse group of authors reflect a growing momentum to make biodiversity research and its application more inclusive and equitable than its historical origins, more prominent in contemporary natural resource management, and reflective of millennia of wisdom gained through long-term relationship with the natural world. The following essay reflects our shared experiences of engaging with and centering Indigenous governance principles, and the values they embody, within the context of our place-based research partnerships.

## 2. Indigenous values, governance principles and laws of the Northwest Coast

In the context of today's global crisis, an examination of the ancient nuučaan̓uł way of life may have something useful to contribute. - Umeek Richard Atleo, 2011 [1, pp. 5–6]

Contemporary challenges of climate disturbance, environmental change, equity, and justice are daunting in their enormity and relatedness, yet they are by no means new. Although their magnitude is unprecedented, Indigenous communities around the world have a long history of responding to extreme climatic events, ecological change and socio-political disruptions [11]. These perturbations to reciprocal relationships between people and place have spurred the development of governance systems and management practices that have supported social-ecological resilience and biodiversity over millennia [12–15]. The values-based principles upon which these governance systems are grounded provide a lens through which to reconsider, rethink and reframe how we approach, define and conduct biodiversity science, conservation and management.

For more than 12 000 years, human societies have been shaping and sustaining diverse landscapes and seascapes across Earth's ecosystems [8,16–18]. In fact, the most biodiverse areas on the planet, often characterized as ‘natural’, ‘intact’ and ‘wild’, are those that have long histories of use and stewardship by Indigenous people [18–20]. This is in part because people have been performing vital ecological functions for millennia, transporting seeds and animals to ensure viable populations [21,22], applying controlled fire to promote biodiversity and landscape heterogeneity [23,24], and terracing and tilling sediment to boost plant and animal production [25], all amid major biophysical disturbances.

Evidence of this abounds along the Northwest Coast of North America. For example, ancient stories and archaeological data reveal that people living along stretches of the Northwest Coast during the Holocene experienced and responded to dramatic sea level rise, at times more than half a metre over the course of one generation [26,27]. At the same time, people were actively cultivating, managing and caring for plants [28] and marine life [29,30]. For example, continued plant translocations and cultivation created biologically diverse and productive ancient forest gardens [20]. Similarly, the construction of intertidal rock-walled terraces created clam gardens known to double clam production [31]. These intertidal innovations required re-engineering to adapt to changing sea-levels [32]. Size and sex selective fishing practices for Pacific salmon, enacted at river mouths with wooden weir and stone trap technologies, sustained salmon productivity and resilience to climatic disturbances for thousands of years [33,34]. Foundational to these Northwest Coast innovations are governance systems and principles that embody explicit values. Governance systems are devised and refined by groups of people over time. As such, they reflect and are grounded in values, that in turn shape beliefs, practices and principles used often or deemed important by those people. Indigenous governance systems of the Northwest Coast centre around principles, laws, protocols and management practices that value the relationships among all forms of life in the natural (humans, plants, animals) and spiritual (ancestors, supernatural beings) worlds, all of which are viewed as kin with distinct roles, rights and responsibilities [1]. In a wide range of ways, these governance systems are acutely attuned to what Western science has come to understand as ‘biodiversity’, and they have been for millennia. Yet Indigenous governance systems permeate all aspects of society: environmental, social, political, economic, constitutional and philosophical [1]. Consequently, they reflect and attend to complexities of biodiversity, including humans as prominent components, and issues of equity and justice simultaneously.

Northwest Coast Nations share similar values that inform governance principles and laws, though each Nation has unique legal and political orders that guide relationships with all life forms in their territories including the management of those relationships by people (table 1; electronic supplementary material, video S1). For Xaayda, these are referred to as Xaayda Kil Yahdas, for nuučaan̓uł Nations they are ḥawiłmis, and for Haíɫzaqv they are Ǧvı̓ḷás, laws of the ancestors. These governance principles and laws (and the societal values they reflect), including respect, responsibility, reciprocity, making things right, interconnectedness, balance, stewardship and seeking wise counsel, among others, are consistent across these coastal Nations. Yet, each Nation has a unique expression of each governance principle in practice (table 1).

**Table 1.** Indigenous governance principles shared among the Xaayda, nuučaan̓uł, and Haíɫzaqv Nations from the Northwest Coast of North America.

governance principles	X̱aayda Kil Yahdas - Haida Laws	nuučaan̓uł *ḥawiłmis* - Nuu-chah-nulth Laws	Ǧvı̓ḷás - Heiltsuk Laws (laws of the ancestors)
respect	*Yahguudang—all acts must be done with respect. We respect each other and all living things*^a^	*ʔiisaak—greater respect. Understanding and accepting differences*^b^	*Xáɫa—all life has equal value. We acknowledge and respect that all plants and animals have a life force*^c,d^
responsibility	*’Laa guu ga kanhllns—we accept the responsibility to manage and care for the land and sea together*^a^	*maamums—your role and responsibilities that match your standing within each Nation. Ex: hereditary leaders have responsibility as caretakers of the land*	*Sála—to be in control of your actions. Intelligent* *behavior to strive for. Thinking of the consequences before you do something*^c,e^
reciprocity	*Isda ad diigii isda—giving and receiving. Reciprocity is an essential practice for interactions with each other and the natural and spiritual worlds*^a^	*hu?aa yii?ap—giving back. Giving and taking*	*giving back goodness received*^c^
make things right/ accountability	*Tll'yahda—make it/things right. If an act is not done with respect or consent, you must make it right*	*caacim ‘high up’—make things right. Make things healthy*	*H̓aíkḷá—to make things right when needed. To make amends*^c^
interconnectedness	*Gina ‘waadluxan gud ad kwaagid—everything depends on everything else*^a^*. All things are connected*	*hišukʔiš c̓awaak—everything is one, everything is interconnected*^b^	*we are all one and our lives are interconnected. Our relationship with our territory is fundamental and we regard it as an extension of ourselves*^d^
balance	*Giid tlljuus—the world is as sharp as the edge of a knife. Balance is needed in our interactions with the natural world*^a^	*q^w^aa?aqλin tiičmis*—*life in balance*	*Nuáqi—one's thoughts. Balance of mind, body, emotions and spirit*^c^
stewardship, to take care	*TllXanda*	*ʔuuʔaałuk—taking care of. Caring and working for next generations*^b^	*cĺísĺá to take care of; h̓íkila—to take good care of something*^c^. *We are steward of the land and sea from which we live, knowing that our health as a people and our society is intricately tied to the health of land and waters*^d^
seeking advice and counsel and sharing knowledge	*Gina k'aadang.nga gii uu tll k'anguudang—seeking wise council. Xaayda elders teach about traditional ways and how to work in harmony with the natural world*^a^	*ḥaaḥuupst̓ał*—*sharing teachings or teaching each other. For teachings to live on they must be taught and re-taught*^b^	*Tq̓ílá—give advice on what to do and how things should be*^c^
land, ocean and people for which a hereditary chief has responsibility and authority to caretake	*Tllgaay* or *Tllga*	*ḥaḥuułi—a Leader's territory which includes ocean, lands, and people for which they have responsibility to caretake*^b^	*ɫáxvái—authority that underpins ǧvı̓ḷás and the strength received from enacting ǧvı̓ḷás*

^a^HlG̱aagilda X̱aayda Kil Ḵ’aalang Skidegate Haida Immersion Program X̱aayda Kil Glossary. 2021; Council of the Haida Nation and Government of Canada. 2018. Guiding principles from the Gwaii Haanas Gina ‘Waadlux̱an KilG̱uhlG̱a Land-Sea-People Management Plan.

^b^Uu-ath-luk 2018. Uu-ath-luk Strategic Plan: building on our successes [35]. Atleo, ER 2011 [1,36].

^c^Heiltsuk Tribal Council. 2018. Dáduqvḷá qṇtxv Ǧvı̓ḷásax̌: To look at our traditional laws. Decision of the Heiltsuk (Haíɫzaqv) Dáduqvḷá Committee regarding the October 13, 2016, Nathan E.Stewart Spill [37].

^d^Brown F, Brown YK. 2009. Staying the course, staying alive. Coastal First Nations fundamental truths: biodiversity, stewardship and sustainability [34].

^e^Reid, C 1988. Heiltsuk (Haíɫzaqv) Cultural Education Center.

**Figure 1.**
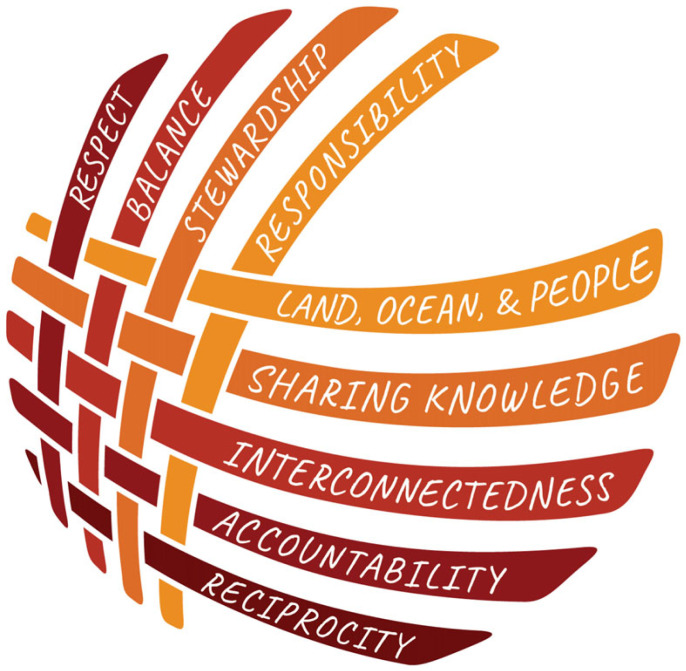
Haíɫzaqv, nuučaan̓uł and Xaayda governance principles are each represented as a strip of cedar bark. These pieces are woven together just as these governance principles are interconnected. Graphic by Arianna Augustine, Stz'uminus Nation, 2023. (Online version in colour.)

Indigenous governance principles and laws are enacted and witnessed in public settings, and their persistence is recounted and preserved in oral histories, narratives and stories [38] (electronic supplementary material, video S1). While such established accounts act as accessible forms of public memory, they are also forms of legal precedent that can be drawn on to legitimately resolve issues in decentralized legal orders and specify management protocols (electronic supplementary material, video S2). Many laws and principles are often embedded in and learned about through stories because Indigenous laws themselves are interconnected and inseparable (figure 1; electronic supplementary material, video S3). While Indigenous oral histories are now recognized as evidence in Canadian courts, they have yet to make their way into judicial reasoning or written in Canadian jurisprudence [38]. Yet momentum is growing to revitalize Indigenous laws and the governance systems in which they are embedded, apply them to pressing biodiversity and environmental issues, and effect change [37,39].

Opportunity exists to learn from and apply these governance principles in the application of biodiversity and conservation science and environmental management. For example, we have the opportunity to: (i) *Tll'yahda* (make things right in Xaayda)—acknowledge historical injustices, overexploitation and past management mistakes, and aim to correct them; (ii) incorporate *hišukʔiš c̓awaak* (interconnectedness in nuučaan̓uł)*—*by considering species interactions, including humans, historical baselines and intergenerational thinking in our study and management of ecosystems; (iii) *Xáɫa*—(respect in Haíɫzaqv) respectfully consider the needs, objectives and rights of diverse species and sectors of society and our *maamums* (responsibility in nuučaan̓uł) to uphold them; and (iv) *Gina k'aadang.nga gii uu tll k'anguudang* (seek wise counsel in Xaayda)—inform decisions based on the best available information from diverse knowledge systems.

## 3. A reckoning of values underlying the colonial history of biodiversity science

For biodiversity researchers and practitioners to learn from and apply Indigenous governance principles today, we first must *Tll'yahda—*acknowledge the history of biodiversity science as an enabler of both colonial expansion and industrial exploitation of natural resources. The sciences of taxonomy, biodiversity and ecology that developed in the eighteenth and nineteenth centuries facilitated the expansion of European colonization of the globe by identifying and cataloguing natural resources (e.g. crops, wildlife, commodities and medicines) and associated knowledge systems of Indigenous people that could also be exploited, exported and used to support empires abroad [40–43]. Scientists look upon this era as one of discovery, scientific inquiry and enlightenment, often without recognizing colonial principles that motivated sciences of the era, and the indispensable and conscious role of naturalists in colonial expansion [42,44–47]. Yet institutions ranging from the Catholic Church to the Royal Society of London and the British Admiralty understood the value of biodiversity science as fundamental to the establishment and success of colonies. Naturalists' exploration and extraction of knowledge from Indigenous peoples abroad fuelled an era of bioprospecting and colonization legitimized by the now-discredited notion of *Terra Nullius* (nobody's land), and the Doctrine of Discovery that were used by Europeans to justify the exploitation and theft of land, Indigenous knowledge and natural resources. In this fashion, pioneers of biodiversity science (e.g. Joseph Banks, Hans Sloane, Alexander von Humboldt, Charles Darwin) and numerous others were instrumental in using nascent sciences of biodiversity for the successful establishment or expansion of colonies in the Americas, Australia, New Zealand and many islands in the Pacific [48–51]. Centuries after this era of colonization, biodiversity research as a colonial practice continues to manifest today in the form of, for example, ‘helicopter science’ [52,53], bioprospecting for extractive industries [54], and research that ignores Indigenous knowledge, intellectual property, and the governance systems in which that knowledge is embedded [55].

While early biodiversity research was used to identify diverse natural resources and knowledge systems that might be exploited, modern natural resource management has historically ignored biodiversity [56,57]. Management approaches in the twentieth century generally omitted both how biodiversity affects resources of interest and how resource exploitation affects biodiversity upon which human communities and other ecosystem constituents depend. Instead, Western resource management historically focused on compartmentalized goals for individual species or species assemblages rather than stewardship of complex ecosystems [58,59]. As a result, many of the scientific models, practices, agencies, laws and technologies used to manage complex systems like fisheries, agriculture, or recovery of endangered species have, until recently, focused on single species objectives. Species interacting within diverse assemblages and complex ecosystems became managed in isolation by centralized and siloed institutions that were geographically removed from the complex systems they were mandated to manage [5,60,61]. Western management driven by values such as efficiency, reductionism, and accumulation of wealth (i.e. maximization of economic rent), and the sciences that support it, are in opposition to, and often at the expense of, the Indigenous values expressed as governance principles such as respect, interconnectedness, balance, and responsibility.

While calls and mandates for ecosystem-based management from Western institutions are decades old [62,63], substantial inertia in the legal doctrines, institutional structures and scientific ‘best practices’ used in fisheries, wildlife, ocean, and landscape management, have precluded their uptake and application [5,64]. In the oceans, it was not until the notable collapse of several of the world's dominant fisheries and degradation of marine ecosystems that high level international advisory panels formed [65,66] and biodiversity science and objectives (in terms of ecosystems, populations, genetic, geographical and even human community diversity) would begin to be applied to marine fisheries in a substantive way.

Since the late 1990s, there have been significant efforts to move towards ecosystem-based management and apply frameworks that aim to incorporate biodiversity in decision-making. Yet, many of these efforts are still predicated largely on ‘stock and flow’ models and economic objectives. For example, the ecosystem services and natural capital approaches often fail to account for reciprocal interactions among various components of ecosystems and contrasting objectives among diverse communities of people, and thereby neglect issues of equity and justice among beneficiaries of said ‘services’ [67–74]. Science, management, and conservation practices are often motivated by a constrained set of Western values; these then continue to perpetuate the process of colonization and undermine the responsibilities of Indigenous peoples to manage their relationship with the land and sea in accordance with their ancestral governance principles. Moreover, these practices ignore broader issues of equity, access and values of both Indigenous and settler societies. This is notably evident in the case of the recovery of sea otters along the Northwest Coast of North America.

## 4. The complex case of sea otter recovery

Evidence of continuous human coexistence with sea otters following the arrival of maritime people to the Americas exists throughout the Holocene in oral histories and the archaeological record [75–77]. Highly valued, hunted, controlled and traded by Indigenous people for at least 12 000 years, it was not until nearly 200 years ago that sea otters were extirpated from the northeast Pacific by the international maritime trade in sea otter fur. Beginning in earnest by the 1750s in Alaska and the 1780s in British Columbia, the commercial trade in sea otter pelts and bioprospecting of the eighteenth and ninteenth centuries led to the introduction of a Western cash economy and the imposition of colonial settlement and laws. This eroded First Nations economies and governance structures that had been in place for millennia. These structures included well established trade networks, spatially explicit marine tenures, and complex traditional resource management protocols and governance principles designed to ensure sustainability [1,13,34,78]. The commercial trade and extirpation of sea otters from the Northwest Coast contributed to the transformation of Indigenous societies and triggered a social-ecological regime shift across much of the northern Pacific Rim [79]. Consequently, the recent recovery of sea otters and their continued range expansions create both challenges and opportunities to reconsider how biodiversity is understood, studied and managed in near shore ecosystems.

### (a) Challenges

As a keystone predator, sea otters can dramatically alter near shore biodiversity, ecosystem structure, primary production and the availability of resources upon which people and other organisms depend [80,81]. On rocky shores, sea otters effectively limit the densities and sizes of herbivorous sea urchins among other macroinvertebrate prey. The loss of sea otters from rocky reef ecosystems can therefore lead to increased urchin grazing and reduction in kelp forests [82,83], while recovery of sea otters has facilitated restoration of diverse assemblages of kelp and other algae [84] which provide shelter, habitat and food for many species of invertebrates and fishes [85,86]. In soft sediment habitats, the physical disturbance associated with sea otter foraging for bivalves and crabs, for example, significantly reduces densities and size of these food sources, but the associated physical disturbance can also increase genetic diversity of seagrass beds or alter food web interactions so as to increase seagrass abundance [87–91].

In one sense, the recovery of this once endangered keystone predator is a remarkable conservation success story, owing in large part to international and national policies that stopped the commercial exploitation and trade of sea otters and protected them from all hunting. Yet when contrasted against historical baselines where sea otter hunting and shellfish harvest were sustained by people over millennia and when viewed through the lens of social justice and food sovereignty today, it becomes an example of a policy failure that has imposed severe inequities among coastal communities. For place-based Indigenous communities, many shellfish species that are consumed by sea otters, as well as fishes that benefit from their indirect effects on kelp habitat, are essential sources of food, micronutrients, medicine, livelihoods, social practices and cultural identity [92]. Prior to the maritime fur trade, Indigenous communities had governance and management protocols in place that secured both the persistence of sea otters and human access to shellfish for food, tools, and trade. Today in Canada, Indigenous peoples have a constitutionally protected right to access shellfish for food, social and ceremonial purposes, but they are not permitted to hunt or scare sea otters as they once did, with the explicit purpose of protecting key shellfish areas. As a result, after decades of sea otter recovery, there are now long stretches of coastline where certain shellfish populations have become so depleted that community members can no longer harvest shellfish locally [93]. Sea otter recovery and range expansion in the Pacific Northwest has therefore introduced significant governance, management, legal and ethical challenges regarding biodiversity [94].

Federal laws, institutions, and scientists in both the United States and Canada are ill-equipped to handle the social, economic and ecological trade-offs associated with the vast direct and indirect ecosystem-wide consequences of unchecked sea otter recovery. Instead, both countries have independent management agencies, laws and paradigms for fisheries, species at risk, and their habitats. As a result, modern management of sea otter recovery in Canada and the United States occurs largely in isolation of fisheries, justice, health and Indigenous sovereignty considerations. Yet, a management decision in any one space, or set of spaces, regarding sea otter recovery, near shore fisheries and Indigenous access rights, affects the others.

### (b) Opportunities

New, spatially nuanced knowledge of sea otter interactions has widened the operating space in which to re-envision how society might meet competing social-ecological objectives associated with the recovery of this keystone species. Studies of sea otter movements and spatial ecology have demonstrated that sea otter populations are structured at small spatial scales [95], and thus regional abundance and distribution is better understood as a mosaic of semi-discrete, localized populations whose density can vary greatly [96,97]. Local scale variation in sea otter abundance, and the strength of their top-down influences, can allow for the emergence of spatial mosaics of kelp and urchin dominated reefs. In some locations, this pattern of contrasting reef habitats, combined with the tendency of individual sea otters to exhibit specialized diets [98,99], leads to increased foraging by otters on energy-rich urchins along the margins of kelp forests and urchin barrens, thereby enhancing the resistance of remnant kelp forests to overgrazing by sea urchins [100]. Moreover, in places where sea otter occupancy is low owing to both hunting by people and the perceived risk of hunting, shellfish prey can recover [101]. Critically, these contemporary findings mirror those found in the archaeological record in multiple areas of the Northwest Coast.

Data from coastal archaeological sites suggests that for much of the mid to late Holocene, sea otters were rare or absent from stretches of coastline where people gathered shellfish. This suggests that spatial mosaics of sea otter presence and absence, and their ensuing indirect effects on kelp habitat, were also a feature of the past coastlines [102]. This spatial patterning may have been one mechanism by which humans and sea otters coexisted for millennia, each prominent predator having its own spatial domain. The revitalization of Indigenous sea otter hunting practices and governance authority to spatially manage the relationships between people, sea otters, shellfish and kelp can enable sea otter and human coexistence again today [92]. Collectively, these lines of evidence highlight the critical consequences of spatially explicit sea otter foraging behaviour, and the prominent role humans played historically, and can play again today, in this complex set of relationships. Importantly, they also chart an established path forward to the possibility of human-sea otter coexistence and regional stability of kelp and shellfish-dominated reef mosaics amid disturbances, like extreme climatic events and disease epidemics.

To confront the complex challenges associated with sea otter recovery, it is essential to equip coastal communities and management institutions with shared and current knowledge, alternative ecosystem-based management strategies and equitable governance structures to navigate the social-ecological regime shifts triggered by the recovery of this keystone predator. The case of sea otter recovery, emblematic of natural resource management issues more broadly, demands innovative models of shared governance based on clear and comprehensive recognition of peoples' values and objectives both in the research process and its application to management. Below we offer an example of how one might apply Indigenous values, governance principles and ancient laws to characterize, monitor and manage biodiversity change associated with sea otter recovery in more inclusive, integrative and equitable ways.

## 5. An Indigenous governance approach to sea otter recovery

Here, our collective understanding of how Haíɫzaqv, nuučaan̓uł and Xaayda governance principles apply to management of sea otter recovery in these Nations’ territories has come through transdisciplinary workshops, listening circles, in-person meetings, online dialogues and decades of research among the authors and other Indigenous scholars, hereditary leaders, and Indigenous knowledge holders. The following discussion reflects our shared experiences of engaging with and centring these governance principles within the context of our place-based, enduring, and evolving research partnerships. We recognize however that our discussion of these governance principles is in its infancy and fails to capture many aspects (e.g. the spiritual and supernatural) that are central to Indigenous ways of knowing and being. We also recognize that these governance principles and our understanding of how they may be applied are embedded in and are inseparable from Haíɫzaqv, nuučaan̓uł and Xaayda worldviews and territories.

### (a) Making things right

Among Haíɫzaqv, nuučaan̓uł, and Xaayda Nations, the mechanism by which people are held accountable for their actions is specified through the law of ‘making things right.’ If an act is done without respect or consent among Xaaydas, one must Tll'yahda (make things right) [103]. This includes publicly acknowledging what was done that was wrong and reaching consensus on how to make it right. Similarly, among the Haíɫzaqv, one must H̓aíkḷá (make amends) if one blunders [37]. This same principle of accountability is captured by the word caacim in nuučaan̓uł, which means to ‘make things right’ and ‘make things healthy’.

In the context of sea otter recovery, and natural resource management more broadly, applying the principle of ‘making things right’ means researchers, managers, and policy makers must acknowledge past transgressions and take actions to correct them. ‘Making things right’ when it comes to sea otter research and its application, means acknowledging the original colonial laws and international industrial-scale trade leading to regional sea otter extirpation. To ‘make things right’ we must acknowledge colonial economic drivers of their population collapse and consider values of nature other than those rooted in capitalism. One way we, the authorship team, have sought to do this is by publishing research that describes the role of colonialism, neoliberalism and capitalism in environmental governance [5].

‘Making things right’ also means acknowledging the colonial dispossession of Indigenous lands and waters, attempted erasure of Indigenous stewardship practices, and erosion of Indigenous governance authority. Canada's *Indian Act* still gives the federal government sweeping powers with regards to First Nations membership, political structures, governance, education and cultural practices. To make progress towards ‘making things right’, all aspects of society, including biodiversity and natural resource research initiatives engaging at the science policy interface, must respect Indigenous sovereignty. Within our research partnerships, we have done this by seeking ʕapaak (free, prior and informed consent in nuučaan̓uł) throughout our research process from decision-making bodies identified by communities. In the case of sea otters specifically, ‘making things right’ also means supporting the revitalization of cultural practices and Indigenous-led stewardship initiatives like sea otter hunting for the purpose of maintaining shellfish harvesting sites [92] and the revitalization of sea gardens to increase the production of shellfish and seaweed, and access to fish [30].

‘Making things right’ also means acknowledging the outcomes of state management systems and current federal laws that fail to account for shifting baselines [104], ecosystem interactions, spatial variation in species recovery and dynamics [105] and Indigenous management objectives [5,74]. For example, a lack of monitoring and export-oriented fisheries policies in the twentieth century enabled and failed to act quickly enough to halt the commercial overexploitation and collapse of northern abalone. It remains listed as ‘endangered’ under Canada's *Species at Risk Act* today. This conservation status, owing to declines that happened under federal management, restricts harvest of this culturally important food by people [106]. Moreover, abalone are managed in isolation of one of its major predators, the recovering sea otter, which is listed as a ‘*species of special concern*’. Hunting sea otters in Canada for the purpose of protecting shellfish like abalone is currently prohibited.

### (b) Interconnectedness

The principle of interconnectedness within the natural world is shared among these Nations. This is captured by hišukʔiš c̓awaak in nuučaan̓uł, meaning everything is one, everything is interconnected [1] and Gina ‘waadluxan gud ad kwaagid in Xaayda, meaning everything depends on everything else [103]. The Haíɫzaqv describe their connection to nature as a fundamental truth such that their relationship with their territory is regarded as an extension of themselves [34]. For research and policy related to sea otters, the application of ‘interconnectedness’ means incorporating species interactions, including humans, in our understanding of species recovery and conservation targets. It also means supporting connections among generations, past, present and future, like supporting intergenerational knowledge transfer of ancestral stewardship practices through culture camps, engaging with local schools and initiatives that support emerging Indigenous stewards. Through our research partnerships, we connect generations of all species by integrating archaeological data, Indigenous knowledge and quantitative ecological and social analyses to broaden our understanding of social-ecological dynamics operating on temporal and spatial scales that are impossible to study in real-time.

In the context of biodiversity science and natural resource management more broadly, applying the principle of ‘interconnectedness’ demands systems thinking. It requires disrupting the culture of reductionism that propelled twentieth century science [107]. For socially relevant research to have meaningful outcomes, the disconnect between science as the producer of knowledge and society as the passive recipient of knowledge will need to be replaced by processes that support the co-design, co-production and co-delivery of knowledge.

### (c) Balance and reciprocity

Balance and reciprocity are foundational values and governance principles that guide how the relationships among people, lands and waters are managed. For the Xaayda, Giid tlljuus (balance) is needed in our interactions with the natural world as is Isda ad diigii isda (giving and receiving) [103]. Specifically, care must be taken to avoid reaching a point of no return and active restoration should be used to restore balance where it has been lost [108]. These same concepts are held by the nuučaan̓uł as qʷaaʔaqƛin tiičmis meaning life in balance (wiicuckum Anne Mack 2022, personal communication) and hu?aa yii?ap [35]. Ǧvı̓ḷás of the Haíɫzaqv specifies the law of Nuáqi, meaning balance of mind, body, emotions and spirit, and the act of ‘giving back goodness received’. Both are foundational principles that are needed to sustain interactions among people and the natural world [37].

With respect to sea otter recovery and natural resource management, the governance principles of ‘balance’ and ‘reciprocity’ means articulating and considering the trade-offs among competing species, management objectives and people's viewpoints given the existence of predator–prey interactions, competition among species for shared resources and differences in people's interests. This translates into managing the *relationships* between species like sea otters, endangered abalone, kelp and people as a collective of interactions rather than on a species-by-species basis. It means restoring the role of humans in coastal food webs as these relationships endured for much of the Holocene. In our research, we explicitly invoke balance and reciprocity by experimenting with and deciphering prevailing ecosystem interactions and accounting for multiple objectives that reflect diverse values rather than the dominant ones. We are also transparent about who benefits from which trade-offs. For example, increased livelihoods associated with sea otter recovery, like jobs from tourism and commercial finfish fisheries [86], do not compensate for lost shellfish food security and food sovereignty among subsistence-based communities [92].

In natural resource management, the practice of ‘reciprocity’ or ‘giving back goodness received’ implies active and intentional stewardship and restoration of lands and waters by people. In terrestrial systems this may include the cultivation of productive berry forests, camas fields and estuarine root gardens via intentional burning and weeding [25]. In coastal marine ecosystems it could include the restoration or creation of clam gardens by terracing and tilling intertidal soft sediment habitat, enriching sediment conditions with crushed shell, and transplanting clams, sea urchins and other species [30]. These examples and the principle of reciprocity are tightly connected to ‘stewardship’ or ‘taking care of’ (table 1; figure 1; electronic supplementary material, video S2).

### (d) Respect and responsibility

Respect and responsibility are foundational governance principles for the Haíɫzaqv, nuučaan̓uł and Xaayda Nations, each with their own word and nuanced meaning. For the Xaayda, Yahguudang (respect) is expected to be given to *all* people and *all* living things [103]. In practice, it means always asking for permisson first, harvesting only what is needed, giving thanks to that which is taken and received, and acknowledging those who behave accordingly. It also includes transparency in discussion and actions. To the Haíɫzaqv, Xáɫa acknowledges that *all* life has equal value and a life force that must be acknowledged and respected [34,37]. The word ʔiisaak, meaning respect among nuučaan̓uł Nations, is practised by respecting all begins as well as the Hawiih's (hereditary leader's) role in the management of their ḥaḥuułi (territory), by being community oriented, and understanding and accepting differences [1,35]. Similarly, the Xaayda must Laa guu ga kanhll; accept the responsibility to manage and care for the land and sea often in collaboration with others [109]. Among the nuučaan̓uł maamums describes one's role and responsibility within the Nation that match your standing. For example, hereditary leaders have responsibility as caretakers of the land.

‘*We both have the right to food*.’ – Kii'iljuus Barbara Wilson, St'awaas Xaaydaga, 2013

Because sea otters are valued and respected as kin under nuučaan̓uł, Xaayda and Haíɫzaqv laws, they like people, hold rights to space and food. Therefore, when it comes to sea otter recovery, the laws of ‘respect’ and ‘responsibility’ in practice translate into ensuring that both people and sea otters have access to enough habitat and food to sustain themselves in perpetuity. Practically, this means not only excluding sea otter from specific shellfish harvesting sites for people via hunting and creating a seascape of fear, but it also means identifying areas where sea otter populations can have free access to a reliable source of food and thrive. The laws of ‘making things right’ and keeping ‘balance’ requires that we enable the co-existence of people and sea otters by intentionally delineating spaces and clear boundaries where both can thrive. Among the Tlingit and Xaayda of Southeast Alaska, sea otters had their own areas ‘on the outside waters' and boundaries between outside and inshore areas were maintained [110].

‘A young man going out to hunt and he looks over, and he says to the [sea] otter bobbing, he says, ‘You better go out to the outside waters, or you are gonna end up as a headman's…headman's headdress!’ … There was an understanding between the Otter People and the people that inhabited an area and they saw them and they talked to them. … We had Otter people. We had Wolf people. Eagle People. We had Raven people. The spirit of all these creatures, the Killer Whale People, there was always an understanding between the people and the other people. And they held their boundaries.’ – Deborah Head, Tlingit, Sháan Séet Craig, Alaska [110, p. 184]

These ancestral laws and practices have been successfully enacted in Sitka, Alaska today, where the localized reduction of sea otters by 70% via hunting and its associated risk caused an increase in sea urchins and decline in kelp that matches the spatial pattern of otter occupancy [101]. The result is a regional spatial mosaic of patches, some dominated by kelp and sea otters, others dominated by sea urchin, a spatial pattern in habitat types that probably existed on the coast for thousands of years [102]. These management practices would have led to habitat diversity that could have buffered the effects of disturbances like extreme climatic events or disease epidemics, thereby enabling productive and resilient social-ecological systems for millennia.

### (e) Seeking wise counsel

Finally, these coastal Nations also share the governance principle of upholding and sharing knowledge. Gina k'aadang.nga gii uu tll k'anguudang means seeking wise counsel among the Xaayda [103]. Through the millennia, elders taught the youth about traditional ways and how to work in harmony with the natural world. In practice today, we employ this principle by working with traditional knowledge and scientific information so that communities can respond to change while upholding culture, values and laws [109]. Among the Haíɫzaqv, Tq̓ílá is to give advice on what to do and how things should be [37].

## 6. Looking forward

With a rapidly changing climate and rampant loss of biodiversity across the planet, there remains substantial debate about how to quantify, understand, manage and conserve biodiversity in the face of numerous threats [71,72]. The governance principles described above for 17 coastal First Nations have historically been excluded from the applied sciences and management associated with biodiversity. We advocate for an alternative approach: broadening who benefits from and participates in sciences of biodiversity by diversifying the values underlying such work. Recently, calls in the biodiversity research and conservation world have been made to shift from or expand current frameworks and practices to include a far more broad and equitable view of what constitutes nature's contributions and who those contributions benefit [72]. In the case of the First Nations represented here, this may occur by centring communities and their expression of values embodied in governance principles at the heart of biodiversity research, conservation and management. These governance principles and the values they reflect are shared by a broader cross section of communities globally, and their formal adoption into biodiversity research and its applications may present a pathway towards more equitable and just science and management practices and outcomes. Yet how does such a shift in principles and values that motivate science and management occur in practice? Solutions, we think, require pluralism in worldviews, scientific methodologies [111], environmental governance [112] and legal systems [37,39,113–115].

No approach for detecting and attributing causes of change in biodiversity can claim independence of human values nor perspectives. Rather, what is measured, how, by whom, and for what purpose not only affects the research but also its outcomes and its applications. The history of Western biodiversity science, and indeed its present practice, remains rooted in colonialism and exploitation. Indigenous and place-based peoples bring deep time knowledge of natural history, phenology, ecosystem connections, dynamic historical conditions and reciprocal relationships with humans. Rather than ignore such knowledge, or extract it for asymmetric gains, we advocate for a more inclusive and introspective process. Transdisciplinary research initiatives co-designed with communities, knowledge-holders and decision-makers offer a venue to engage in and learn from diverse worldviews, values, epistemologies and types of data and knowledge. Biodiversity research conducted in true collaboration with communities and in accordance with their values and/or governance principles has the potential to generate both better science and more equitable and just scientific processes and outcomes. Importantly, these approaches involve jointly framing issues and knowledge gaps, and require building relationships based on trust, mutual respect and long-term commitment. Democratizing the research process by way of knowledge co-design, co-production and co-delivery lends legitimacy to the science and knowledge that is produced, empowers local communities and decision-makers, increases societal relevance of the work, and can lead to new perspectives and discoveries [116,117]. We contend that locally rooted biodiversity and observation networks that are co-designed and co-produced from conception through implementation will be better equipped to detect and inform global biodiversity change with increased legitimacy and societal relevance. However, systems of science, knowledge and power are not easily shifted and co-production processes can reflect the institutional (colonial) settings in which they are embedded [118,119].

For biodiversity conservation and natural resource management, there is a need to move from centralized, singular approaches to those that can incorporate a plurality of values, principles and ways of knowing [120,121]. Co-management (or co-governance) is one context in which diverse values regarding biodiversity can be reconsidered. For instance, co-management arrangements (i.e. the sharing of responsibility and decision-making authority among Indigenous governments and state entities within a defined context) are often sites of knowledge ‘co-production’ and shared learning about conservation and management issues [122]. In many jurisdictions, such processes have reduced conflict because they create space for dialogue, require (often formally) inclusion of Indigenous knowledges and identification of shared interests. Still, many co-management arrangements are premised on the logics of colonialism (i.e. ministerial approval and regulatory oversight), are responding to conditions created by colonialism in the first place (e.g. dispossession of resources), and hinge on dominant forms of science (models, single-species, economic optimization) and scientific hegemony [61].

Another approach exists, in which management and science are decentralized into community-based and Indigenous-led approaches. Rather than accommodating colonial institutions, this latter approach requires, at least in part, either their deconstruction, the explicit devolution of their authorities and responsibilities and/or their willingness to accommodate pluralism in legal orders. It also requires that current colonial institutions support, fund and incentivize science, management and governance of biodiversity that is shaped by rather than extracted from Indigenous communities. Examples of legal pluralism and Indigenous resurgence [123] are currently being enacted along the Northwest Coast with the explicit purpose of protecting biodiversity [124]. For example, Indigenous protected areas [125], and their management plans, are being established based on local Indigenous legal principles and governance systems [39], as are environmental impact assessments [37]. Fortunately, there are shared principles of learning and adjusting perspectives in both science and Indigenous governance where expanded knowledge and historical depth regarding the role Indigenous peoples had in managing these systems can now be included in legal orders and policies.

Regardless of the arrangement among groups, there are many ways to accommodate diverse values and governance principles into the study, conservation and management of biodiversity. To be effective in the face of global crises, those of us working at the nexus of biodiversity science, management, and policy cannot remain agnostic to the plurality of values and ways of knowing that permeate societies around the world. We need to critically evaluate biodiversity science initiatives and the conservation and management decisions they aim to support, against all three dimensions of justice, including *distributive justice*—who benefits? *procedural justice*—who is included in the science and decision-making processes? and *recognitional* justice—which groups' responsibilities and rights are recognized [126]? Regardless of what systems we study, biodiversity and natural resource management science and application will benefit from a greater focus on the governance principles of respect, responsibility, reciprocity, interconnectedness, balance, stewardship, seeking wise counsel and making things right among all forms of life.

## Data Availability

The data are provided in the electronic supplementary material [127].
